# Effect of tanniferous leaf meal based multi-nutrient blocks on feed intake, hematological profile, immune response, and body weight changes in *Haemonchus contortus* infected goats

**DOI:** 10.14202/vetworld.2015.572-579

**Published:** 2015-05-06

**Authors:** Surender Singh, A. K. Pathak, R. K. Sharma, Muzaffer Khan

**Affiliations:** Division of Animal Nutrition, Faculty of Veterinary Sciences & AH, Sher-e-Kashmir University of Agricultural Science and Technology of Jammu, R. S. Pura-181 102, Jammu and Kashmir, India

**Keywords:** condensed tannins, goats, *Haemonchus contortus*, leaf meal mixture, multi-nutrient blocks

## Abstract

**Aim:**

The aim was to assess the effect of multi nutrient block (MNB) supplementation with and without tanniferous leaf meal mixture on feed intake, hematological profile, immune response, and body weight changes of goats that were experimentally infected with *Haemonchus contortus*.

**Materials and Methods:**

Total 12 adult male goats of similar age and body weight (26.49±0.87) were allocated in 3 groups in completely randomized design. MNB supplemented in first two groups i.e. in T_1_ (no infection) and T_2_ (*H. contortus* infection @ 1500 L_3_/goat) group, while, MNB-condensed tannin (CT) supplemented in T_3_ (*H. contortus* infection @ 1500 L_3_/goat + CT source). All goats were fed concentrate mixture @ 100 g/day/goat, *ad lib* wheat straw and MNB or MNB-CT to meet their requirement for maintenance. Body weights were recorded and blood and fecal samples were collected at 0 day and thereafter at 15 days intervals for a period of 75 days for the assessment of body weight changes, hematological profile and *H. contortus* loads. Both humoral and cell-mediated immune (CMI) response were assessed at the end of feeding trial.

**Results:**

Mean hemoglobin and packed cell volume (PCV) levels were found to be highest (p<0.001, p<0.05) in T_1_ group followed by T_3_ group and lowest values were observed in T_2_ group. However, The PCV values between T_1_ and T_3_ groups were found to be statistically non-significant (p<0.05). The humoral and CMI response were significantly (p<0.036) higher in T_3_ group as compared to T_2_ group. MNB-CT supplementation significantly (p<0.001) reduced fecal egg counts in T_3_ group as compared to MNB supplemented T_2_ group.

**Conclusion:**

Supplementation of MNB-CT could be used as an alternative sustainable method to control *H. contortus* and maintained health status and performance of goats in face of parasitic challenge.

## Introduction

Goat keeping is an attractive enterprise for farmers in many developing countries. Due to its low rearing cost, good economic returns, well adapted in the existing situations and their ability to thrive on locally available alternative feed resources, goat farming under intensive and semi-intensive system for commercial production, emerging favorable market conditions, and easy accessibility to improved goat technologies has been gaining momentum. However, most of the developing countries of the world lie within the limits of the production constraints caused by scarcity of good quality feeds and fodders and gastrointesti nal nematode (GIN) infections are major hindrance to small ruminant production, specifically in the tropical and subtropical regions of the world. Large economic losses can result from reduced body weights, reduced animal production (milk, meat, skin, fiber, and manure), and ultimately reduced animal health and performance in general [1].

The GINs, predominantly *Haemonchus contortus* pose a severe threat to sheep and goats, especially those having a poor nutritional status [2-5]. The usual mode of control of these GIN based on the repeated use of anthelmintics, however, evolution of resistance against the most commonly used anthelmintics [6-10] and public concern over drug residues excreted in milk and meat products and their potential risk as environmental contaminants [11] and has a negative impact on their use and alternative approaches for control are needed.

Naturally acquired *H. contortus* infection may cause progressive dramatic fall in packed cell volume (PCV) and hemoglobin (Hb) or may remove a fifth of the circulating erythrocyte volume per day from lambs and on average one tenth of the volume over the course of nonfatal infections lasting a few months [12]. The pathogenic effects result from the inability of the host to compensate for blood loss. If the blood loss is small and the host can adequately compensate for the loss, no measurable illness occurs. However, if the rate of blood loss exceeds the host’s hematopoietic capacity a progressive anemia leads to death [13,14]. An increasing number of recent studies indicate that nutrition could affect GIN infections not only through quantitative variations of different diet components, but also by the presence of some qualitative compounds (Condensed tan nins [CT]) in plants consumed by ruminants. The CT might interfere directly with the biology of various developmental stages of GINs [4,15-19]. On the other hand, indirectly, CT could also improve the host nutrition by protecting the dietary proteins from ruminal degradations and this could modulate worm biology [20]. Therefore, the key priority for increasing animal productivity is to ensure that the digestion of poor quality crop residue and the proportion between protein and energy in absorbed nutrients are adequate.

Nutritional modulation of infected animals in order to improve the host resistance and/or resilience to GIN infections seems to represent one of the most promising options to reduce the dependence on conventional chemotherapy and to favor the sustainable control of GIN infections [21]. The supplementation strategy is practical and more appropriate in Indian subcontinent where the treatment costs are too high to afford. To achieve this target, the alternative nutritional strategies should be developed and these should include feed supplementation by use of tanniferous leaf meal mixture (LMM) containing multi nutrient blocks (MNB)-CT. The low cost MNB-CT supplements can enhance the animal’s ability to utilize the available diet and assist the animal to withstand GIN infection with resultant substantial increase in productivity. The MNB-CT combines these three concepts, ensuring anthelmintic efficacy, balanced supply of nutrients, and improves the utilization of poor quality crop residues in animals [22-25].

The ease of preparation and maintenance make the MNB-CT block technology practicable for non-chemical parasites control strategies, organic food production, and for adoption by landless and marginal farmers. The effectiveness of CT to control GINs had been widely investigated worldwide. However, the studies pertaining to the effect of CT containing LMM incorporated MNB supplementation in *H. contortus* infected goats are scarce. Therefore, the present study was conducted to investigate the effect of MNB-CT on feed intake, hematological profile, immune response, and body weight changes in goats infected with *H. contortus* infection.

## Materials and Methods

### Ethical approval

Permission of the Institutional Animal Ethics Committee was taken prior to the start of the experimental study.

### Tanniferous tree leaves

Locally available *Eugenia jambolana* and *Psidium guajaba* branches were harvested in the month of March-April, 2012 from Faculty premises R. S. Pura, Jammu which is subtropical region of J and K state. Tree leaves were separated from its branches and air dried in the shed for 15-20 days. Once the tree leaves were dried, they were processed for preparation of leaf meal by grinding in electric grinder. The leaf meals were stored in large perforated nylon bags in a dry place away from direct sunlight for experimental study.

### Block manufacturing

Two types of MNB involved in this study were prepared by cold process. The proportion of ingredients in the MNB and MNB-CT were 35.0, 0.0, 33.0, 7.25, 7.0, 7.75, 4.0, 4.5, 1.5, and 19.5, 16.5, 33.0, 8.5, 6.0, 7.0, 4.0, 4.0, 1.5%, for mustard oil cake, LMM, molasses, urea, lime stone powder, di-calcium phosphate, mineral mixture, wheat bran, and common salt, respectively. The suitable proportions of wheat bran, mustard oil cake, and urea etc., in MNB were replaced with LMM of *E. jambolana* and *P. guajava* dried leaves by making iso-caloric and iso-nitrogenous. The LMM as CT source was only added in MNB-CT blocks at 16.5% level. In MNB, all processed solidified ingredients were well-mixed and homogenized with liquid mixture composed of water dissolved urea and molasses while in CT containing MNB-CT, LMM of *E. jambolana*, and *P. guajaba* in the ratio of 70:30 which was incorporated as CT source and alternative functional feeds by replacing costlier conventional feed ingredients and then added to solid component which were well mixed and homogenized with above mentioned liquid mixture in a balanced proportion. The properly mixed homogenous semisolid (2.5 kg) material of both types of blocks was then put in a manually operated multi nutrient brick making machine. The blocks were air dried in the shed for 10-15 days until they are hard enough for easy handling, transport, hanging, and for licking of the experimental animals.

### Experimental animals, diet, design, and management

Twelve local adult male goats (26.49±0.87 kg initial average body weight) were divided randomly into three equal groups in a completely randomized block design. MNB supplemented in first two groups i.e. in T_1_ (no infection) and T_2_ (*H. contortus* infection @ 1500 L_3_/goat) group, while, MNB-CT supplemented in T_3_ (*H. contortus* infection @ 1500 L_3_/goat + CT source). All goats were fed concentrate mixture (CM) @ 100 g/day/goat, *ad lib* wheat straw (WS) and MNB or MNB-CT to meet their requirement for maintenance. Feeding trial was conducted for duration of 75 days excluding 30 days of adaptation on MNB feeding and other management practices before the start of experimental feeding and experimental *H. contortus*. The metabolism trial of 6 days excluding 3 days of adaptation was conducted at the end of feeding trial. Digestible crude protein (DCP) was determined by metabolism trial. The DCP was calculated by subtracting the CP excreted through feces from CP intake. To determine total digestible nutrient (TDN), the percentage of each nutrient was multiplied by the coefficient of digestibility for that nutrient and added together. Therefore, the TDN was the sum of DCP, digestible crude fiber, digestible nitrogen free extract, and digestible ether extract multiplied by 2.25. The dietary schedule of goats among three groups was depicted in the Table-1. Nutrient requirements for maintenance of all experimental goats were met as per NRC [26].

**Table-1:** Dietary schedule of goats under different groups.

Particulars	T_1_	T_2_	T_3_
Goats	4	4	4
WS	*Ad lib*	*Ad lib*	*Ad lib*
CM (g/d)	100	100	100
MNB	*Ad lib*	*Ad lib*	-
MNB-CT	-	-	*Ad lib*

*(Maize: 30; wheat bran: 22, mustard oil cake: 45, mineral mixture: 2 and common salt: 1), MNB-CT=Multi nutrient blocks condensed tannins, MNB=Multi nutrient blocks, CM=Concentrate mixture, WS=Wheat straw

All goats were kept in a well-ventilated shed having concrete floor throughout the experiment. Hygienic and sanitary conditions were provided in the shed by routinely using disinfectant and detergent. All management conditions *viz*. deworming, vaccination, etc. were followed as per standard procedures.

### Experimental *H. contortus* infection

Adult female *H. contortus* parasites of caprine origin were collected from the nearest slaughterhouse. The eggs were retrieved from the gravid uterus as per requirement. The infective 3^rd^ stage larvae (L_3_) were produced by petridish method of fecal culture technique. The infective doses of the 3^rd^ stage larvae of *H. contortus* were prepared and administered orally @ 1500 larvae per goat of T_2_ and T_3_ groups.

### Laboratory analyses

The dry matter (DM) content of MNB, MNB-CT, CM, and WS was determined by drying the samples at 100±5°C for 24 h. Kjeldahl nitrogen analyses [27] were performed in triplicate on feeds and CP calculated as (N × 6.25), ether extract content by Soxhlet apparatus and ash content by combustion at 500- 550°C in muffle furnace. Neutral detergent fiber and acid detergent fiber (ADF) was determined by the Van Soest *et al*. [28] method. Condensed tannin content was analyzed by the butanol-Hcl method as per Makkar [29]. The Hb concentration in blood was estimated by cyanomet Hb method. The PCV was estimated by Wintrobe tube method and results were expressed in percent [30].

### Body weight changes

At the start of the experiment, goats were weighed for two consecutive days to get their average initial body weight. The weight of the individual goat was recorded at fortnightly intervals in the morning before feeding and watering for whole experimental period in order to assess the change in body weight.

### Blood collection

Blood samples were collected from all experimental goats at the start of the experiment (0 day) and thereafter at fortnightly intervals. About 2 mL of blood was collected aseptically from the jugular vein using 18-gauge needle in a glass vial containing Ethylene di-amine tetra acetate for hematological studies. Immediately after blood collection the tubes were gently rotated between palms in order to mix it with an anticoagulant.

### Immune response

Humoral immune response was assessed through hemagglutination (HA) titer method [31] with slight modifications. Sheep red blood cells (SRBC) were used as antigen. For immunization, 5 mL of 20% SRBC suspension was injected subcutaneously in all goats of each group on 55^th^ day of the experiment and same feeding schedule was continued till the end of experiment. A booster dose was injected at 10^th^ day of first injection. On day 21^st^ following SRBC injection, bleeding was done. The antibody production in response to immunization with SRBC was assessed by micro HA test. The reciprocal of highest dilution of serum causing complete HA was taken as HA titer of the serum sample and expressed in log_2_ values.

The cell-mediated immune (CMI) response was assessed through *in vivo* cutaneous delayed-type hypersensitivity (DTH) reaction against phytohemagglutinin-p (PHA-p). Toward the end of experimental feeding trial of 75 days, individual goats from each group were used for DTH test. All the goats were injected intra-dermal with 125 µl of PHA-p working solution on one side of the neck region. The thickness of the skin was subsequently measured at 12 h and then 24 h interval up to 96 h.

### Fecal egg counts (FECs)

Fecal samples of all the experimental goats were collected at the start of the experiment and thereafter at fortnightly intervals till the end of the feeding trial. The fecal samples were collected directly from the rectum early in the morning before feeding and watering. The FECs were estimated by using Stoll’s egg counting technique.

### Statistical analysis

All the statistical procedures were done as Snedecor and Cochran [32]. The results obtained were subjected to analysis of variance and treatment means were ranked using Duncan’s multiple range test. The periodic alterations in body weight, hematological parameters, and CMI response were analyzed using repeated measures design (General linear model; GLM, Multivariate). Significance was declared at p<0.05 unless otherwise stated.

## Results and Discussion

The chemical composition of CM, WS, MNB, and MNB-CT is given in Table-2. The CT content of MNB-CT was 1.76%. The chemical composition of CM and WS used in the experiment was comparable with the values reported by many workers [4,33-35].

**Table-2:** Chemical composition of feedstuffs (% DM except DM).

Attributes	CM	WS	MNB	MNB-CT
DM	90.69	92.00	86.93	87.72
OM	93.60	90.53	77.56	78.62
CP	21.62	3.19	34.69	34.47
CF	8.37	38.69	1.82	2.87
Ether extract	3.33	0.76	0.69	0.95
Neutral detergent fiber	0.00	82.52	13.73	13.98
Acid detergent fiber	0.00	53.27	4.38	6.69
CT	0.00	0.00	0.00	1.76

CM=Concentrate mixture, WS=Wheat straw, MNB=Multi nutrient block, MNB-CT=Multi nutrient blocks condensed tannins, DM=Dry matter, OM=Organic matter, CP=Crude protein, CF=Crude fiber, CT=Condensed tannins

The proportions and ingredients composition used for the preparation of MNB in the present study were contradictory to those used by previous workers [36,37]. It might be due to the availability and quality of local feed ingredients. Many workers used cement @ 10-15% as a binder for the preparation of MNB [38], while in this study both types of MNB (MNB and MNB-CT) were prepared without cement.

### Nutrient intake and plane of nutrition

Daily nutrient intakes and plane of nutrition by goats among three different groups are presented in the Table-3. Daily intakes (g/d) of CM, MNB, MNB-CT and WS intakes were found to be statistically non-significant (p<0.05) among T_1_, T_2_, and T_3_ groups. Similar trends were also recorded in daily intakes (g/d) of DM, organic matter (OM), digestible OM, and TDN irrespective of all three groups. However, DCP intake (g/d) was significantly higher in T_1_ group as compared to T_2_ group, while, T_3_ group has an intermediate position between T_1_ and T_2_ groups. Present results are in ­agreement with the findings of Scharenberg *et al*. [39], who also reported non-significant difference in total intake of DM, OM, and ADF in *H. contortus* infected lambs fed on diet with and without tanniferous sainfoin. The CT intake (% of DMI) from MNB-CT was 0.22 in T_3_ group. The nutrient intakes in terms of DM, DCP and TDN (g/d) were remained within the normal range for goats [26] and this clearly indicates both type of supplements (MNB and MNB-CT) along with WS and concentrate for experimental goats were palatable. Present findings suggesting that CT supplementation (as in MNB-CT) at low to moderate level without any adverse effect on DM intake are in consistency with the earlier reports [35,40]. Nutrient density (%) of composite diets in term of DCP and TDN differed significantly (p<0.05) irrespective of groups (T_1_, T_2_, and T_3_). In the present study, plane of nutrition was not affected adversely with MNB-CT supplementation and inconsistency with the earlier reports [35]. Dutta *et al*. [41] reported that voluntary feed intake of *H. contortus* infected lambs increased with increasing DCP than those fed lower protein diets. Present study also revealed that DCP and TDN intake was higher in T_3_ group.

**Table-3:** Effect of MNB and MNB-CT on nutrient intake (g/d) and plane of nutrition by goats during metabolism trial.

Attributes	Group*			SEM	p

	T_1_	T_2_	T_3_		
DM	634.16	599.41	627.34	12.39	0.524
OM	568.15	536.13	561.31	11.05	0.504
CP	58.17	58.43	61.33	1.03	0.416
Digestible CP	38.20^b^	32.48^a^	34.88^ab^	0.96	0.029
Digestible OM	345.67^b^	300.99^a^	329.36^ab^	8.39	0.073
Total digestible nutrient	362.95^b^	316.04^a^	345.82^ab^	8.81	0.073
Concentrate	90.69	90.69	90.69	0.00	
Wheat straw	476.10	437.00	457.70	11.11	0.392
MNB	67.37	71.72	78.95	2.41	0.137
Nutrient density (%)					
Digestible CP	6.02^b^	5.42^a^	5.57^a^	0.10	0.018
Total digestible nutrient	57.29^b^	52.69^a^	55.14^ab^	0.71	0.010
CT % of DMI	0.00	0.00	0.22	0.03	0.000
Fecal egg counts (per gram of feces)					
0^th^ day of infection	-	-	-	-	-
21^st^ day of infection	-	187.50^b^	37.50^a^	39.81	0.000
75^th^ day of infection	-	1162.50^b^	237.50^a^	198.43	0.000

ab Means with different superscripts within a row differ significantly (p<0.05),

* T_1_=Negative control, T_2_=Infected control, T_3_=Infected treatment, %=Percent, CT=Condensed tannins, MNB=Multi nutrient block, MNB-CT=Multi nutrient blocks condensed tannins, DMI=Dry matter intake, g/d=Gram per day, SEM=Standard error of mean, OM=Organic matter, CP=Crude protein

### Body weight changes

**Figure-1:**
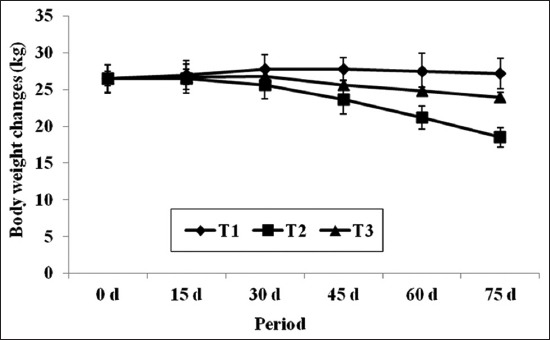
Effect of multi-nutrient block and multi-nutrient block condensed tannin on body weight changes in *Haemonchus contortus* infected goats.

The body weight changes of experimental goats for a period of 75 days were presented in Figure 1. Initial body weights (kg) of goats did not differ significantly (p<0.05) irrespective of groups, however, the mean body weight at the end of experiment was significantly (p<0.001) higher in T_1_ as compared to T_2_ group, while T_3_ groups has an intermediate value between both T_1_ and T_2_. As the time period of *H. contortus* infection increased body weight declined significantly (p<0.001) in both infected groups (T_2_ and T_3_) as compared to negative control (T_1_) group. Body weight changes when compared between both infected groups, significantly (p<0.035) reduced body weight was recorded in T_2_ as compared to T_3_ group. Present results conflicted with the findings of previous work ers [4,42] who reported that there was no significant changes in the body weight of sheep infected with *H. contortus* having tanniferous plant (*Prosopis cineraria*) and tanniferous LMM in their diet in comparison to infected control. A significant amount of protein is redirected for repair of damaged tissues, synthesis and production of immunoglobulin and reducing the amount of protein deposition in the muscles. This leads to reduction of body weight in T_2_ and T_3_ as compared to T_1_ group. However, MNB-CT supplementation gives an indication that binding effect of CT was pronounced at lower level by supplying protein to the lower gut and subsequently it’s more efficient use for tissue bear and tears [35,40].

### Hematological profile

**Figure-2:**
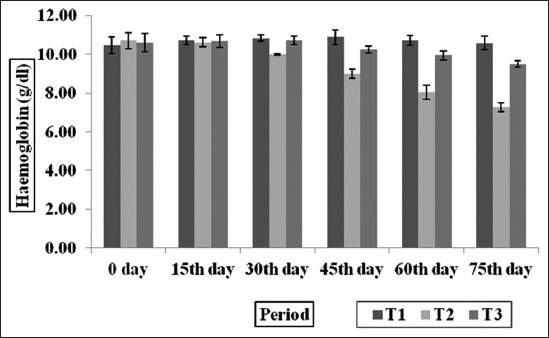
Effect of condensed tannins containing multi-nutrient blocks on hemoglobin in *Haemonchus contortus* infected goats.

**Figure-3:**
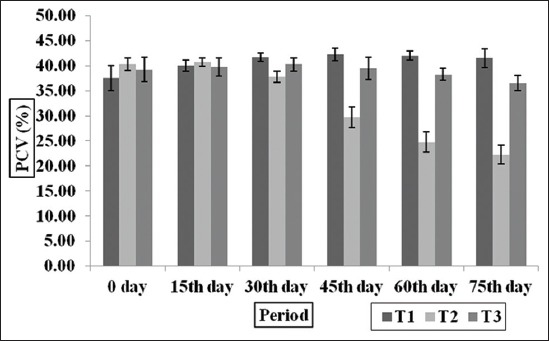
Effect of condensed tannins containing multi-nutrient blocks on packed cell volume in *Haemonchus contortus* infected goats.

The Hb and PCV values of experimental goats among three groups and at different time intervals are depicted in the Figures-2 and 3. The Hb (g/dl) was found to be highest (p<0.001) in T_1_ followed by T_3_ group and lowest Hb was observed in T_2_ group. The PCV values were significantly (p<0.001) higher in T_1_ and T_3_ groups as compared to T_2_ group, however, the PCV values between T_1_ and T_3_ groups did not differ significantly. The Hb and PCV values were within the normal physiological range suggested for goats [43]. This suggests that the general health of goat’s supplemented MNB-CT remained satisfactory throughout the experiment. The observation indicated that low CT level in MNB-CT as an additive and induced no adverse effect on target parameters like Hb and PCV in *H. contortus* infected goats. This is in agreement with the finding of Dey *et al*. [35]; Dubey *et al*. [44] and Pathak *et al*. [4] who reported that 0-2% CT of the diet in lambs and kids during 6 months feeding trial. The Hb and PCV were maintained in normal physiological range in T_3_ group as compared to T_2_ group, which could be due to the lesser intensity of blood-sucking *H. contortus* parasites and their FECs in T_3_ group.

### Humoral immune response

**Figure-4:**
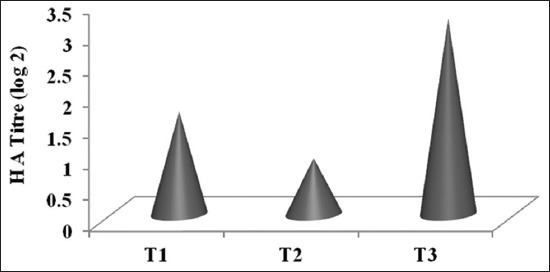
Effect of condensed tannins containing multi-nutrient blocks supplementation on humoral immune response in goats against sheep red blood cells.

Humoral immune response study indicated beneficial effects of CT supplementation through MNB-CT in infected goats. Mean serum HA titers (log2) against SRBC were presented in the Figure 4. There was a significant (p<0.05) effect on humoral immune response against SRBC in experimental goats of T_3_ as compared to T_1_ and T_2_ groups. Hence, the humoral immune response against SRBC was significantly (p<0.05) highest in T_3_ which was offered MNB-CT block and the present finding is supported by Niezen *et al*. [45] and Min *et al*. [46]. Similar to the present finding Kumar *et al*. [47] observed that the birds given tannins containing raw red sorghum exhibited higher humoral immune response assessed through HA titer. By making the protein unavailable for digestion and absorption until it reaches the more acidic abomasum, CT also enhance nutrition by providing high-quality protein to the small intestines. This high-quality protein bypass effect has the potential to enhance the immune response and increase resistance to GIN [48]. The positive response of the immune system to protein intake has been shown in metabolism studies with sheep [40], lambs [49], and kids [50]. Bypassing amino acids like arginine, glutamine, and cysteine can enhance immune responses as these amino acids regulate activation of T and B lymphocytes, lymphocyte proliferation, and the production of antibodies [51]. The increase in availability of essential amino acids might have contributed to the improved humoral immune response observed in present study.

### CMI response

**Figure-5:**
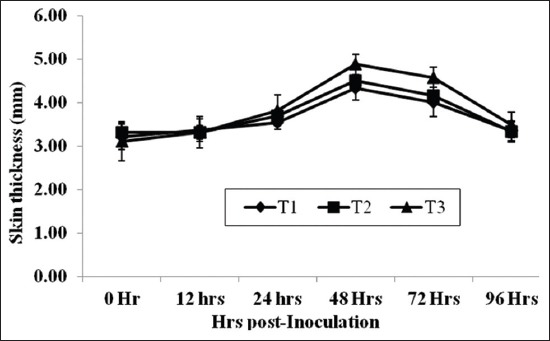
Delayed-type hypersensitivity response to phytohemagglutinin-p in goats on condensed tannins containing multi-nutrient blocks supplementation.

The CMI response measured as *in vivo* DTH response to PHA-p in all experimental goats have been presented in the Figure 5. Mean values for skin thickness differed significantly (p<0.036) among different groups and the maximum (p<0.036) skin thickness was observed in T_3_ group as compared to T_2_ group. However, the mean value of skin thickness (mm) in T_1_ group has an intermediate position between T_2_ and T_3_ groups. Skin thickness was increased significantly (p<0.001) from 24 h post inoculation onward, however, the maximum skin thickness was recorded on 48 h and 72 h post-inoculation of PHA-p and then gradually trim down up to 96 h. Therefore, the goats of MNB-CT supplemented group (T_3_) showed significantly higher CMI response to PHA-p as compared to MNB supplemented both groups (T_1_ and T_2_), which might be attributed to the higher availability of sulfur containing amino acids at abomasal and small intestine level. Lower CT level in feeds given to animals has been reported to increase non-ammonia nitrogen flux to the small intestine, to increase the absorption of essential amino acids. It seems that CT may have some stimulatory effect to the immune system of the animals [20,40]. The significant benefits of MNB-CT supplementation in goats were ascertained as a protein protectant, natural anthelmintic and immunomodulator.

### FECs

At the beginning of the experiment each goat gave zero egg counts, however, after 21 days of experimental *H. contortus* infection in T_2_ and T_3_ groups showed passing parasitic eggs in their feces. Though, FECs were zero in goats of T_1_ group throughout the experimental period, so they were not included in the statistical analysis. Mean FECs were significantly (p<0.001) higher in T_2_ group as compared to T_3_ group (Table-3). The FECs in T_2_ group were increased significantly (p<0.001) throughout the study periods (75 days) relative to their counterparts in T_3_ group. Reduced FECs could be attributed to direct effect of CT on fecundity, killing of adult worms, and indirectly by improving immune function against GIN through enhanced in tissue protein supply [45]. Alternatively, the CT could form a complex with nutrients and inhibit nutrients availability for larval growth or decrease GINs metabolism directly through inhibition of oxidative phosphorylation [52], causing larval death [53]. Pathak *et al*. [54,55] have reported that CT extracts from various tree leaves can disrupt the life cycle of *H. contortus* by preventing their eggs from hatching and by preventing larval development to the infective stage. The results of present study are in agreement with the previous reports [4,40], who reported that CT supplementation may be used as an alternative parasite management strategy. Both direct and indirect effects of CT against *H. contortus* infection appear to be occurring in the present study.

## Conclusion

It may be concluded that the supplementation of MNB-CT blocks have a noticeable encouraging impact on body weights, hematological profile, and immune response in *H. contortus* infected goats without affecting nutrient intake and utilization. Therefore, this may be the better option as alternative feed supplement/functional feeds and natural dewormer for controlling *H. contortus* infection in goats as socioeconomic, eco-friendly sustainable approach.

## Authors’ Contributions

SS was the MVSc Scholar of the division who carried out experimental research work and laboratory analysis of data. AKP was guide of SS, under whose supervision, the thesis was submitted. AKP planned and designed the experiment; formulated condensed tannins enriched multi nutrient blocks, executed statistical analysis of data, drafted and revised the manuscript as per journal format. RKS was head of the division. MK helped in preparation of multi nutrient blocks and assisted in sample analysis in the laboratory. All authors read and approved the final manuscript.
